# Association of free fatty acid binding protein with central aortic stiffness, myocardial dysfunction and preserved ejection fraction heart failure

**DOI:** 10.1038/s41598-021-95534-1

**Published:** 2021-08-13

**Authors:** Chih-Hsuan Yen, Jiun-Lu Lin, Kuo-Tzu Sung, Cheng-Huang Su, Wen-Hung Huang, Yun-Yu Chen, Shih-Chieh Chien, Yau-Huei Lai, Ping-Ying Lee, Yen-Yu Liu, Jui-Peng Tsai, Cheng-Ting Tsai, Charles Jia-Yin Hou, Ying-Ju Chen, Yu-Jou Hsieh, Chung-Lieh Hung, Ta-Chuan Hung, Hung-I. Yeh

**Affiliations:** 1 Division of Cardiology, Department of Internal Medicine, MacKay Memorial Hospital, No. 92, Chung-Shan North Road, 2nd Section, Taipei, 100 Taiwan; 2 Mackay Junior College of Medicine, Nursing and Management, Taipei, Taiwan; 3 Institute of Epidemiology and Preventive Medicine College of Public Health, National Taiwan University, Taipei, Taiwan; 4 Department of Medicine, MacKay Medical College, New Taipei City, Taiwan; 5 Division of Endocrinology and Metabolism, Department of Internal Medicine, MacKay Memorial Hospital, Taipei, Taiwan; 6 Heart Rhythm Center and Division of Cardiology, Department of Medicine, Taipei Veterans General Hospital, Taipei, Taiwan; 7 Department of Critical Care Medicine, MacKay Memorial Hospital, Taipei, Taiwan; 8 Critical Care Medicine, Department of Internal Medicine, Mackay Memorial Hospital, Tamsui, Taiwan; 9 Department of Telehealth, Mackay Memorial Hospital, New Taipei City, Taiwan; 10 Institute of Biomedical Sciences, Mackay Medical College, New Taipei City, Taiwan

**Keywords:** Cardiology, Medical research

## Abstract

There is an established link between cardiometabolic abnormality, central arterial stiffness, and preserved ejection fraction heart failure (HFpEF). Adipocyte free fatty acid binding protein (a-FABP) has been shown to signal endothelial dysfunction through fatty acid toxicity, though its role in mediating ventricular-arterial dysfunction remains unclear. We prospectively examined the associations of a-FABP with central arterial pressure using non-invasive applanation tonometry (SphygmoCor) and cardiac structure/function (i.e., tissue Doppler imaging [TDI] and global longitudinal myocardial strain [GLS]) in patients with cardiometabolic (CM) risk (n = 150) and HFpEF (n = 50), with healthy volunteers (n = 49) serving as a control. We observed a graded increase of a-FABP across the healthy controls, CM individuals, and HFpEF groups (all paired p < 0.05). Higher a-FABP was independently associated with higher central systolic and diastolic blood pressures (CSP/CPP), increased arterial augmentation index (Aix), lower early myocardial relaxation velocity (TDI-e′), higher left ventricle (LV) filling (E/TDI-e′) and worsened GLS (all p < 0.05). During a median of 3.85 years (interquartile range: 3.68–4.62 years) follow-up, higher a-FABP (cutoff: 24 ng/mL, adjusted hazard ratio: 1.01, 95% confidence interval: 1.001–1.02, p = 0.04) but not brain natriuretic peptide, and higher central hemodynamic indices were related to the incidence of heart failure (HF) in fully adjusted Cox models. Furthermore, a-FABP improved the HF risk classification over central hemodynamic information. We found a mechanistic pathophysiological link between a-FABP, central arterial stiffness, and myocardial dysfunction. In a population with a high metabolic risk, higher a-FABP accompanied by worsened ventricular-arterial coupling may confer more unfavorable outcomes in HFpEF.

## Introduction

Heart failure (HF) with preserved ejection fraction (HFpEF, left ventricular ejection fraction ≥50%) accounts for nearly 50% of all patients with HF and is drastically increasing in the global aging society. Patients with higher cardiometabolic (CM) risks are at particularly higher risk of HFpEF^1–3^. Arterial stiffness, though modifiable with intensive exercise, remains common co-morbid conditions in subjects presenting CM disorders, and have shown to be a crucial factor in the pathophysiology of diastolic dysfunction and HFpEF^1,4–6^. The application of central hemodynamic index of arterial stiffness using non-invasive tonometry either at rest or specific maneuvers (e.g. postural changes or during exercise) may serve as sensitive marker of vascular aortic function, which likely better reflects the true volume and pressure load on the heart over peripheral arterial measures^7–10^.

Several circulating adipocytokines and neurohormonal activities in relation to a variety of CM disorders may contribute to endothelial dysfunction and arterial stiffness^11–13^. Interestingly, higher circulating adipocyte free fatty acid-binding protein (a-FABP) tightly related to several CM disorders (e.g. metabolic syndrome or type 2 diabetes) has been reported to elicit oxidative stress through activated cytokines leading to increased vascular stiffness^13–16^. Furthermore, both clinical and experimental studies reported that a-FABP is widely involved in cardiovascular diseases, adverse cardiac remodeling and HF development^16–18^. As a-FABP has shown to modulate adverse cardiac metabolism in subjects manifesting CM disorders, higher a-FABP has been reported to contribute to HFpEF through suppressed myocardial function^16–19^. Nevertheless, it remains unknown that whether a-FABP may directly and adversely affect central arterial stiffness in subjects manifesting increased CM risk, a presumed key regulator in the pathogenesis of HFpEF^1,9^.

To this end, we aimed to investigate the association of a-FABP with central hemodynamic information and myocardial function in HFpEF among subjects manifesting higher cardiometabolic risk, and further explore the combined use of a-FABP with central arterial stiffness measures in HFpEF outcomes.

## Methods

### Study population and design

In this prospective study, we enrolled 254 consecutive study participants from outpatient clinics in a single tertiary medical center from December 2011 to September 2014. More detailed study design, setting, and exclusion criteria were published previously^20^. In brief, our study subjects comprised the following three groups: a healthy control group, including healthy volunteers who underwent an annual health survey with no known cardiovascular or systemic diseases; a high CM risk group, including patients with at least one known CM risk factor; and a HFpEF group, including patients carrying at least 1 CM risk with history of HF hospitalization and preserved left ventricle ejection fraction (LVEF > 50%). CM risk factors included hypertension (HTN), type 2 diabetes (DM), dyslipidemia, obesity (*>* grade 1, body mass index [BMI] ≥ 30 kg/m^2^), and central obesity (sex-specified abnormal waist circumference > 90 cm in men or > 80 cm in women) Comprehensive echocardiography, biochemical laboratory data, and several pro-inflammatory/HF biomarkers were examined. Traditional blood pressure measurements were recorded at the brachial artery using a standardized sphygmomanometer device after adequate rest in the sitting position for at least 15 minutes. Patients with atrial fibrillation, moderate to severe valvular heart disease, or systolic heart failure were excluded from this study.

Clinical characteristics, hemodynamic information, and central hemodynamics on arterial pressure tracing via tonometry were all obtained. This study was conducted in accordance with the Good Clinical Practice Guidelines by the Institutional Review Board, with written informed consent obtained from all study participants. This study was approved by the institutional review board of Mackay Memorial Hospital (approval number: 11MMHIS127; 15MMHIS031e). In the current study, we defined the pre-specified clinical endpoints with prospective follow-up. The primary endpoint was HF hospitalization after the study index date following the completion of central hemodynamics information and a-FABP collection on the same day. Our secondary study endpoint was set to explore the associations of a-FABP with various central hemodynamic indices, echocardiography-derived parameters, and the incidence of HF hospitalization. We continued to follow the participants’ clinical events until the end of March 2019.

### Assessment of central and peripheral aortic hemodynamic waveforms and stiffness

**Figure 1:**
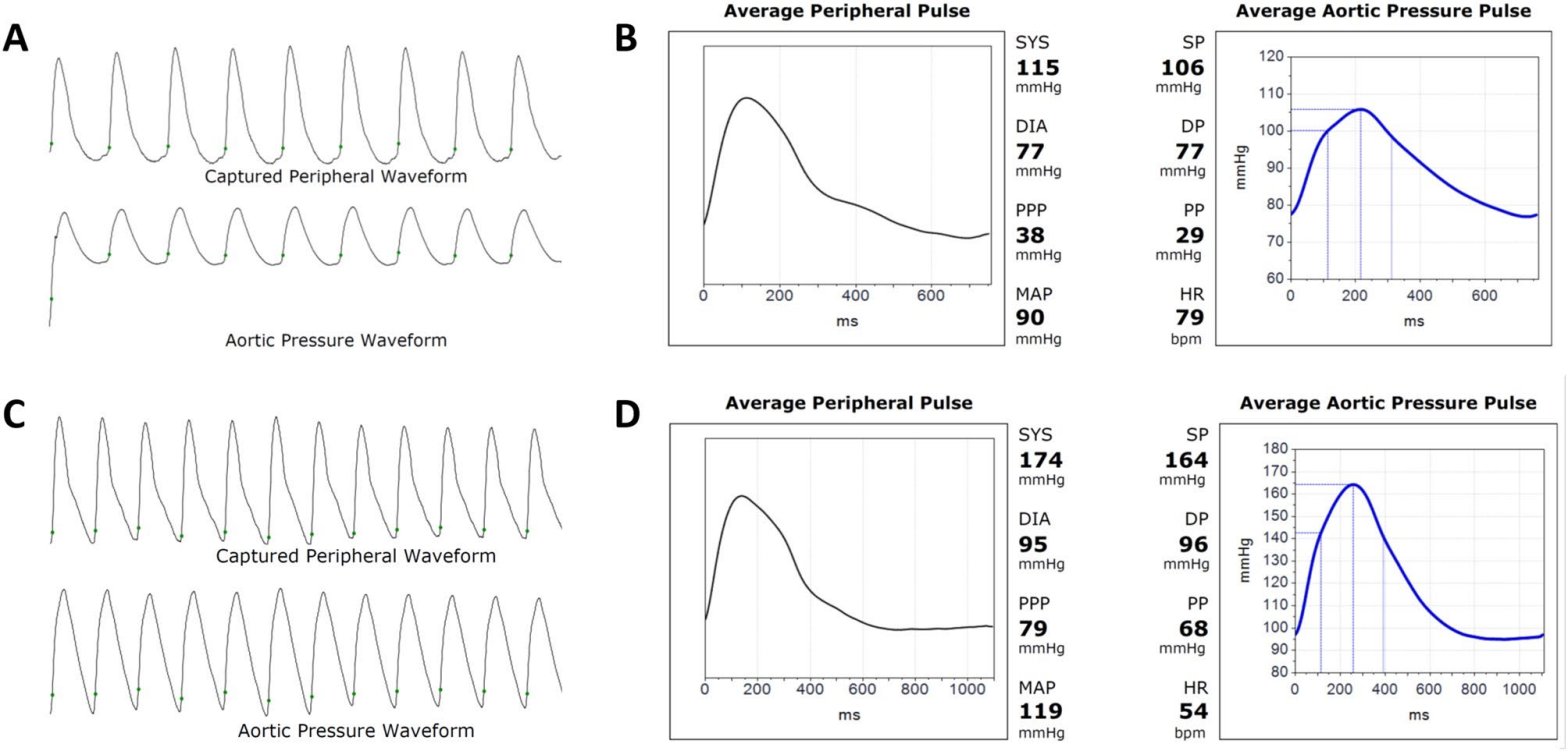
Representative waveform analyses in patients with and without heart failure. (**A**) Waveforms illustrating peripheral (radial) and central (ascending aorta) hemodynamic measurements in a 58-year-old female patient without cardiometabolic risks or heart failure. (**B**) Corresponding detailed waveform analysis of the same patient in (**A**). (**C**) Waveforms of peripheral and central hemodynamic measurements in a 64-year-old female patient with heart failure with preserved ejection fraction. (**D**) Corresponding detailed waveform analysis of the same patient in (**C**).

Central aortic hemodynamic and stiffness indices were acquired using the well-validated, non-invasive method of applanation tonometry (SphygmoCor, AtCor Medical, Sydney, Australia) (version number: 1.3.1.4; URL link: https://atcormedical.com/technology/sphygmocor-xcel/) at the radial artery after peripheral blood pressure acquisition from the brachial artery^21^. In brief, waveforms from the ascending aorta, including CSP and CDP, were derived using the transfer function and a commercial software and device (SphygmoCor 9; AtCor Medical, Sydney, Australia) (as Fig. 1)^22^. Peripheral (brachial) blood pressure was also measured in duplicate during the central waveform acquisition and used to calibrate radial waveforms. Afterwards, peripheral pulse pressure (PPP) and CPP were calculated as the difference between paired systolic blood pressure (SBP) and diastolic blood pressure (DBP) or CSP and CDP on the peripheral and central waveforms in each study subject, respectively, with peripheral pulse pressure amplification (PPA) calculated as a ratio of PPP/CPP^23^. Aortic augmentation index (AIx), a composite marker reflecting systemic arterial stiffness and LV afterload status measured by pulse wave amplification (PWA), was calculated using augmented pressure (AP) as a percentage of the total central pulse pressure (AP/CPP) from baseline^22,24^. Regional (central) arterial stiffness was assessed by aortic pulse wave velocity (PWV) using electrocardiogram (ECG)-gated sequential tonometry at the carotid and femoral sites.

### Cardiac structure and function: diastolic indices and global LV strain

Two-dimensional echocardiography equipped with a 2.5–4.0 MHz transducer (Vivid 7, GE, Vingmed, Norway) was performed to determine the conventional cardiac structure and function. The cardiac structure, including ventricular wall thickness, dimensions, and derived LV mass, was assessed using the linear method as recommended by the American Society of Echocardiography^25^. Doppler-based early mitral inflow E wave (E), late A wave (A), derived E/A ratio, and iso-volumetric relaxation time were all obtained following the standardized protocol. Tissue Doppler based myocardial imaging (TDI), including peak myocardial systolic velocity (TDI-s′) and early diastolic relaxation velocity (TDI-e′) from both septal and lateral basal segments, were determined using high frame rate pulsed-wave Doppler imaging techniques.

LV strain, a novel dimensionless myocardial contractility metric, was determined using sophisticated software (version 10.8, EchoPAC, GE Vingmed Ultrasound, Norway). The detailed imaging acquisition protocol and analysis algorithm has been described previously^26^. In brief, the LV endocardial border was carefully manually traced from three LV apical views utilizing 2D images with an average acquired frame rate of 60–80 frames per second (fps): four- (4CH), two- (2CH) and three chamber (3CH) at the LV end-diastolic phase. The software automatically generated an epicardial LV silhouette and a region of interest (ROI) comprising six sub-segments in each apical view after selecting landmark points, followed by automated segmental tracking. After tracking, a wave representing the longitudinal systolic myocardial strain (LS) was displayed throughout the whole cardiac cycle. For statistical ease, LS was expressed as absolute values. In this regard, higher absolute values of LS represent better LV contractility. Global LV longitudinal systolic myocardial strain (GLS) values were derived from averaged LS of 3 LV apical views (4CH, 2CH, and 3CH) and served as an indicator of global myocardial systolic function for each individual patient.

### Laboratory measurements and examination of biomarkers

We analyzed several standard laboratory markers including blood glucose levels, lipid profiles, renal functions (e.g. estimated glomerular filtration rate (eGFR)), brain natriuretic peptide (BNP), and a-FABP. Venous blood samples were collected from participants by a trained study nurse after adequate fasting and sent for analyses in a central laboratory. The concentrations of BNP, galectin-3, N-terminal pro-peptide of type III procollagen (PIIINP), and a-FABP (catalog number RD191036200; BioVendor, Inc., Czech Republic) were determined using commercially available enzyme-linked immunosorbent assay kits.

### Statistical analysis

Data are expressed as mean ± standard deviation for normally distributed continuous variables and as proportions for categorical variables. Continuous variables were analyzed using a two-tailed t-test. Discrete variables were compared using a Chi-square test. Missing data were omitted from the analysis. Backward stepwise regressions were used to explore the relationships between several central hemodynamic measures, baseline clinical co-variates, and a-FABP, with parameters with p > 0.1 excluded from the models. Associations of a-FABP with various echocardiography parameters and central hemodynamic components were further examined using multi-variate linear regression models.

Outcome analyses were conducted using a Cox regression hazard model to determine the risk of incident HF, including: (1) Model 0: crude effect; (2) Model 1: adjusting for age and gender; (3) Model 2: adjusting for age, gender, BMI, HTN, diabetes, coronary artery disease, stroke, eGFR, and LV mass index; (4) Model 3: Model 2 plus hyperlipidemia and smoking status. The event-free survival curves were plotted using the Kaplan–Meier method with a log-rank test to assess their statistical differences. Time-dependent receiver operating characteristic (ROC) curves and area under the curve (AUC) statistics (as Harrell's C-index) were used to determine the prognostic performance of parameters to predict HF events, with optimal threshold value (cutoff point) of parameters in predicting HF events calculated using the Youden index of AUC (maximum of sensitivity + specificity − 1). A stratified outcome-driven analysis integrating information about central hemodynamic indices and biomarkers of BNP or a-FABP was conducted, assessing the possible effects of biomarkers on central hemodynamics outcome prediction.

Statistical significance was set as p < 0.05. The analyses were performed with SAS^®^ software (version 9.4, SAS Institute Inc., Cary, NC, USA) and STATA (StataCorp. 2015. *Stata Statistical Software: Release 14*. College Station, TX: StataCorp LP) software.

## Results

### Patient characteristics

**Figure 2:**
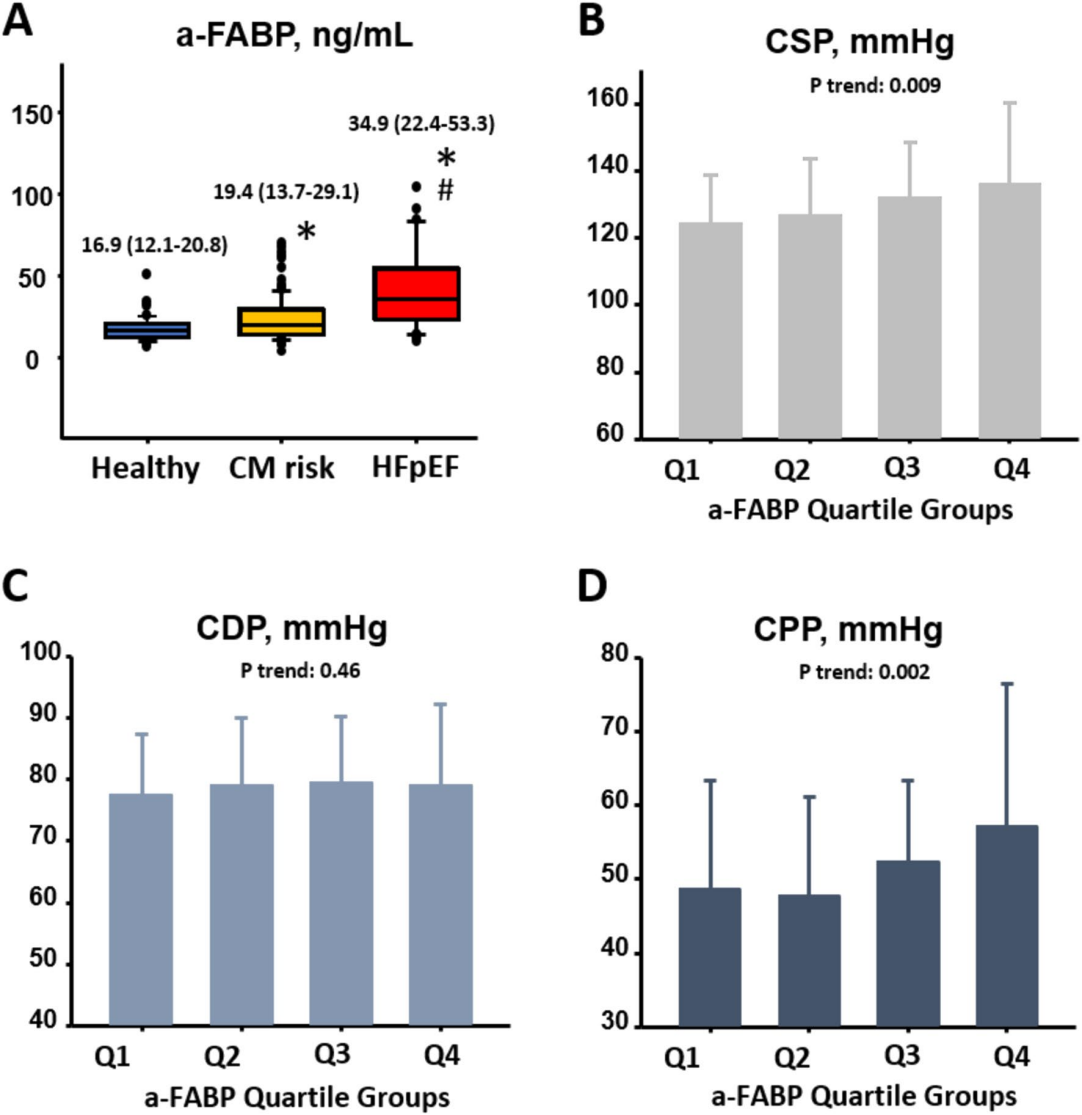
Central hemodynamic pressures of each patient group. (**A**) Significant and graded increase of a-FABP level across study participants classified as healthy, with cardiometabolic risk (CM) and HFpEF categories. *p < 0.05 vs healthy group; ^#^p < 0.05 vs CM group. (**B**–**D**) Central hemodynamic measures of CSP, CDP and CPP across a-FABP quartiles. a-FABP range (ng/mL): Q1: < 13.9; Q2: 13.9–19.9; Q3: 19.9–30.3; Q4: > 30.4. The CSP and CPP increased as the a-FABP increased (both p for trend: < 0.05). Adipocyte free fatty acid binding protein (a-FABP).

Patients who experienced a clinical HF event were older, or had a larger waist, a greater BMI, both a higher systolic pressure and a higher pulse pressure, DM, HTN, hyperlipidemia, higher fasting glucose, lower HDL-c, worse renal function (higher BNP), markedly higher a-FABP, or prior HF history (Table 1). A significantly greater LV wall thickness, greater LV mass, more prolonged deceleration time, larger iso-volumic relaxation time, lower LV TDI-e′, higher E/TDI-e′, lower TDI-s′, and worsened GLS were also associated with clinical HF events (Table 1). Finally, significantly higher CSP, CDP, CPP and AIx were observed in patients who experienced HF events (Table 1). By categorizing study participants into healthy, known CM risk, and HFpEF as 3 groups, we observed that a-FABP was significantly higher in the HFpEF group (median: 34.9, interquartile range [IQR 22.4–53.3] ng/mL) compared to the CM risk group (median: 19.4 [IQR 13.7–29.1] ng/mL) and healthy group (median: 19.4 [IQR 13.7–29.1] ng/mL); with higher a-FABP observed in the CM risk group compared to that in the healthy group (all paired p < 0.05) (Fig. 2A). Higher BMI was associated with higher a-FABP (r = 0.30, p < 0.001) in the current study.

**Table 1:** Baseline patient characteristics.

Variables	HF event (−)	HF event (+)	P value
Total (N = 249)	(N = 191)	(N = 58)	
**Baseline demographic information**			
Age (years)	64.2 ± 9.6	71.0 ± 8.9	< 0.001
Sex, women (%)	63.4	72.4	0.2
Heart rate (1/min)	67.3 ± 9.8	69.0 ± 11.3	0.27
Systolic blood pressure (mmHg)	137.8 ± 19.5	149.3 ± 18.8	< 0.001
Diastolic blood pressure (mmHg)	80.1 ± 11.9	83.2 ± 13.2	0.09
Pulse pressure (mmHg)	57.7 ± 12.8	66.1 ± 14.0	< 0.001
Waist circumference (cm)	88.5 ± ± 12.0	94.2 ± 10.4	0.002
Body mass index (kg/m^2^)	26.1 ± 4.4	28.0 ± 4.0	0.003
**Medical history**			
Diabetes (%)	20.4	50	< 0.001
Coronary artery disease (%)	8.4	13.8	0.22
Stroke (%)	2.1	1.8	0.87
Hypertension (%)	67	86.2	0.005
Prior HF history (%)	10	55.2	< 0.001
Medications for hyperlipidemia (%)	41.4	60.3	0.01
Active smoking (%)	11	16	0.35
Fasting sugar (mg/dl)	110.2 ± 35.3	125.1 ± 44.3	0.008
Triglyceride (mg/dl)	119.1 ± 70.4	142.9 ± 115.9	0.07
HDL-c (mg/dl)	57.0 ± 20.1	48.4 ± 13.7	0.003
eGFR (ml/min/1.73 m^2^)	83.0 ± 24.2	65.2 ± 27.1	< 0.001
**Biomarkers**			
BNP (pg/ml)	37.0 ± 72.4	131.1 ± 201.2	< 0.001
a-FABP (ng/mL) (n = 240)	22.2 ± 12.3	42.9 ± 35.9	< 0.001
**Cardiac structure and geometry**			
Septal wall thickness (mm)	9.0 ± 1.3	9.9 ± 1.8	< 0.001
Left ventricular ejection fraction (%)	67.1 ± 5.06	67.8 ± 7.57	0.33
Left ventricular mass index (gm/m^2^)	76.8 ± 16.3	84.8 ± 20.9	0.003
**Cardiac function**			
Deceleration time (ms)	216.1 ± 49.7	236.5 ± 56.3	0.009
Iso-volumetric relaxation time (ms)	87.8 ± 17.7	95.4 ± 16.9	0.004
TDI-e′ (average) (cm/s)	8.1 ± 1.9	6.6 ± 1.7	< 0.001
E/TDI-e′ (average)	9.1 ± ± 3.0	12.3 ± 4.2	< 0.001
TDI-s′ (average) (cm/s)	7.8 ± 1.4	7.1 ± 1.2	0.001
GLS (%)	− 20.2 ± 2.2	− 18.3 ± 2.2	< 0.001
**Peripheral hemodynamic indices**			
Pulse pressure amplification (PPA) (ratio)	1.23 ± 0.34	1.18 ± 0.41	0.37
**Central hemodynamic indices**			
Heart rate during central hemodynamics	67.3 ± 9.8	69.0 ± 11.3	0.27
Aortic augmentation index (AIx) (%)	20.6 ± 9.8	24.3 ± 9.9	0.02
Central systolic blood pressure (CSP) (mmHg)	127.6 ± 17.7	143.1 ± 23.3	< 0.001
Central diastolic blood pressure (CDP) (mmHg)	77.6 ± 11.5	82.4 ± 12.2	0.007
Central pulse pressure (CPP) (mmHg)	50.0 ± 15.3	60.8 ± 17.6	< 0.001

HF, heart failure; LDL-c, low-density lipoprotein-cholesterol; HDL-c, high-density lipoprotein-cholesterol; eGFR, estimated glomerular filtration rate; BNP, brain natriuretic peptide; a-FABP, adipocyte free fatty acid binding protein; LV, left ventricle; E/A ratio, E wave/late A wave of Doppler-based mitral inflow; TDI-e′, peak myocardial diastolic relaxation velocity on tissue-based Doppler; E/TDI-e′, E wave/TDI-e′; TDI-s′, peak myocardial systolic relaxation velocity of tissue-based Doppler; GLS, global LV longitudinal systolic myocardial strain.

### Associations of a-FABP with central hemodynamics

By using backward stepwise regression, we observed that advanced age, HTN (coefficient: 7.16, 95% confidence interval (CI) 1.59–12.75, p = 0.01), higher a-FABP (coefficient: 2.05, 95% CI 0.47–3.63, p = 0.01, per + 10 ng/mL), and higher triglycerides were independently associated with higher CSP. Instead, higher triglycerides, HTN (coefficient: 5.73, 95% CI 2.33–9.13, p = 0.001), and male sex (coefficient: 6.12, 95% CI 3.02–9.22, p < 0.001) were independently associated with CDP. Advanced age (coefficient: 3.39, 95% CI 1.39–5.38, p = 0.001, per 10-year increment), female sex (coefficient: 8.09, 95% CI 4.09–12.09, p < 0.001), higher triglyceride, and higher a-FABP (coefficient: 1.66, 95% CI 0.3–3.02, p = 0.017, per +10 ng/mL) were independently associated with CPP. Finally, HTN (coefficient: 4.13, 95% CI 0.81–7.44, p = 0.015), female sex (coefficient: 6.47, 95% CI 3.81–9.67, p < 0.001), presence of coronary artery disease (coefficient: 4.70, 95% CI 0.45–8.95, p = 0.03) and lower eGFR (coefficient: − 0.71, 95% CI − 1.21 to − 0.22, p = 0.005, per − 10 ml/min/1.73 m^2^) were independently associated with AIx. Only the presence of HTN (coefficient: 0.11, 95% CI 0.005–0.22, p = 0.04) was independently associated with PPA. These associations were confirmed using forward stepwise regression analyses. Using a univariate linear regression model, higher a-FABP was independently associated with all central hemodynamics. After multivariate adjustment in full models, higher a-FABP was independently associated with higher CSP (coefficient: 2.00, 95% CI 0.78–3.21) and CPP (coefficient: 1.42, 95% CI 0.45–2.39, both p < 0.05), but not CDP, AIx or PPA (all p = NS) (Table 2).

**Table 2:** Associations of a-FABP with central hemodynamic indices and key cardiac structural and functional parameters.

(Per 10 ng/mL a-FABP increment)	Uni-variate model		Multi-variate model 1		Multi-variate model 2	
	Coef. (95% CI)	p value	Coef. (95% CI)	p value	Coef. (95% CI)	p value
**Central and peripheral hemodynamic indices**						
CSP (mmHg)	3.05 (1.60, 4.51)	0.001	2.72 (1.23, 4.20)	< 0.001	2.00 (0.78, 3.21)	0.001
CDP (mmHg)	1.45 (0.68, 2.21)	0.01	1.08 (0.19, 1.97)	0.018	0.57 (− 0.13, 1.28)	0.11
CPP (mmHg)	2.35 (1.16, 3.54)	< 0.001	1.59 (0.73, 2.45)	< 0.001	1.42 (0.45, 2.39)	0.004
PPA (ratio)	0.001 (− 0.03, 0.03)	0.90	0.005 (− 0.03, 0.04)	0.77	0.002 (− 0.004, 0.01)	0.50
AIx (%)	0.85 (0.25, 1.45)	0.006	0.64 (0.05, 1.24)	0.035	0.37 (− 0.27, 1.01)	0.26
**Cardiac structure and function**						
Left ventricular mass index (gm/m^2^*)	2.23 (− 0.54, 1.80)	0.001	2.07 (1.06, 3.09)	< 0.001	1.20 (0.14, 2.25)	0.026
TDI-e′ (average) (cm/s)	− 0.26 (− 0.37, − 0.15)	< 0.001	− 0.17 (− 0.27, − 0.07)	0.001	− 0.11 (− 0.22, − 0.01)	0.036
E/TDI-e′ (average)	0.69 (0.40, 0.97)	< 0.001	0.34 (0.15, 0.53)	< 0.001	0.20 (0.003, 0.40)	0.046
TDI-s′ (average) (cm/s)	− 0.12 (− 0.20, − 0.04)	0.005	− 0.07 (− 0.15, 0.01)	0.089	− 0.02 (− 0.11, 0.06)	0.59
GLS (%)	0.30 (0.17, 0.44)	< 0.001	0.27 (0.14, 0.41)	< 0.001	0.18 (0.04, 0.33)	0.013

*In which model body mass index was not entered. **Multi-variate Model 1:** Adjusted for age, gender; **Multi-variate Model 2**: Model 1 plus body mass index (BMI), hypertension (yes/no), diabetes mellitus (yes/no), active smoking status (yes/no), coronary artery disease (yes/no), stroke (yes/no), renal function (eGFR).

AIx, aortic augmentation index; CSP, central systolic pressure; CDP, central diastolic pressure; CPP, central pulse pressure; GLS, global LV longitudinal systolic myocardial strain; PPA, peripheral pulse pressure amplification; TDI-e′, peak myocardial diastolic relaxation velocity on tissue-based Doppler; E/TDI-e′, mitral inflow E wave divided by TDI-e′; TDI-s′, peak myocardial systolic relaxation velocity of tissue-based Doppler.

### Associations of a-FABP with cardiac structure and function

Overall, we observed that higher a-FABP was associated with more unfavorable LV remodeling, lower peak myocardial systolic velocity (TDI-s′) and early diastolic relaxation velocity (TDI-e′), higher LV filling E/TDI-e′, and a more impaired global LV longitudinal strain (GLS) measure (Table 2). In fully adjusted models, we observed independent associations among higher a-FABP and higher LV mass index (coefficient: 1.20, 95% CI 0.14–2.25, p = 0.026), lower TDI-e′ (coefficient: − 0.11, 95% CI − 0.22 to − 0.01, p = 0.036), higher E/TDI-e′ (coefficient: 0.20, 95% CI 0.003–0.40, p = 0.046) and worsened GLS (coefficient: 0.18, 95% CI 0.04–0.33, p = 0.013) (Table 2). The associations of central hemodynamics with cardiac structure and function were detailed in Supplemental Materials (including Supplemental Table 1).

### Effects of central hemodynamics, biomarkers, and the risks of incident HF events

**Figure 3:**
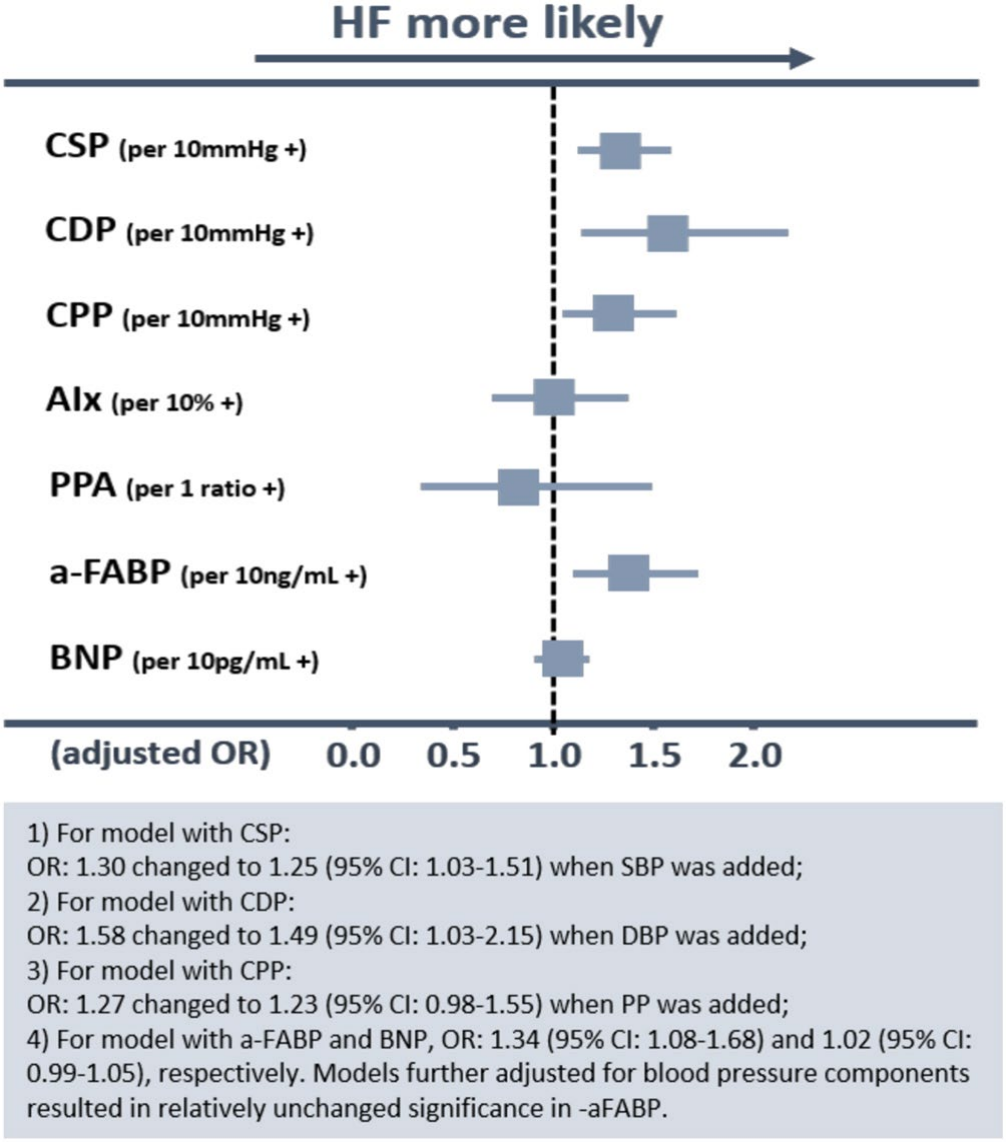
Predictors of heart failure hospitalization. When history of heart failure (HF) was incorporated into the models, all central hemodynamic indices, except AIx, and a-FABP remained as independent predictors for incident HF. Models were adjusted for clinical co-variates as: age, sex, body mass index, and known history of heart failure, hypertension, diabetes, coronary artery disease, hyperlipidemia medication use, and renal function (eGFR). Abbreviations as Table 1.

During a median follow-up of 3.85 years (IQR 3.68–4.62 years), a total of 58 HF events occurred (LVEF: 50.8–74% for patients with event). a-FABP alone showed an AUC of 0.67 (95% CI 0.60–0.73) in the prediction of incident HF with an optimal cutoff set at 24.0 ng/mL by Youden index (0.35). Prognostic performance by central hemodynamics in HF incidence and individual optimal cutoffs from respective Youden index further detailed in Supplemental Figure 1. Using univariate Cox regression analysis, all central hemodynamic measures, a-FABP, and BNP were all strongly correlated with HF events (Table 3). The HF predictive powers of CSP and CDP were not modified by either a-FABP or BNP (data not shown), except for a marginal effect between CPP and a-FABP (p interaction: 0.06). When prior history of HFpEF was incorporated into the models, all central hemodynamic parameters and a-FABP, rather than BNP level, remained as independent predictors for incident HF (Fig. 3A). Interestingly, greater BMI modified the negative prognostic impact of a-FABP on incident HF even after multivariate adjustment (p _interaction_: 0.007), with those presenting with larger body size and higher a-FABP more likely to experience a HF event.

**Table 3:** Predictors of future HF hospitalizations.

Variables	Model 0		Model 1		Model 2		Model 3	
	HR (95% CI)	P value	HR (95% CI)	P value	HR (95% CI)	P value	HR (95% CI)	P value
**Biomarkers**								
a-FABP (ng/ml)	1.015 (1.009–1.020)	< 0.001	1.049 (1.006–1.018)	< 0.001	1.010 (1.000–1.019)	0.04	1.010 (1.001–1.019)	0.04
BNP (pg/ml)	1.002 (1.001–1.003)	< 0.001	1.002 (1.001–1.003)	< 0.001	1.002 (1.000–1.003)	0.01	1.002 (1.000–1.003)	0.01
**Hemodynamic information**								
CSP (mmHg)	1.022 (1.012–1.033)	< 0.001	1.019 (1.009–1.030)	< 0.001	1.016 (1.005–1.027)	0.004	1.016 (1.005–1.027)	0.005
CDP mmHg)	1.019 (0.997–1.037)	0.087	1.025 (1.004–1.047)	0.02	1.023 (1.000–1.047)	0.046	1.025 (1.001–1.049)	0.04
CPP (mmHg)	1.029 (1.016–1.042)	< 0.001	1.023 (1.009–1.037)	0.001	1.019 (1.005–1.034)	0.01	1.019 (1.004–1.034)	0.01

Variables with a p values < 0.10 in Model 0 were selected into **Model 1–Model 3** for evaluating adjusted effects.

**Model 0**: Crude effect; **Model 1:** Adjusted for age, gender; **Model 2**: Model 1 plus body mass index (BMI), hypertension (yes/no), diabetes mellitus (yes/no), coronary artery disease (yes/no), stroke (yes/no), renal function (eGFR), left ventricular mass index; **Model 3**: Model 2 plus hyperlipidemia and active smoking status (yes/no).

CI, confidence interval; HFpEF, heart failure with preserved ejection fraction; HR, hazard ratio; eGFR, estimated glomerular filtration rate; a-FABP, adipocyte free fatty acid binding protein; BNP, brain natriuretic peptide; CSP, central systolic pressure; CDP, central diastolic pressure; CPP, central pulse pressure.

**Figure 4:**
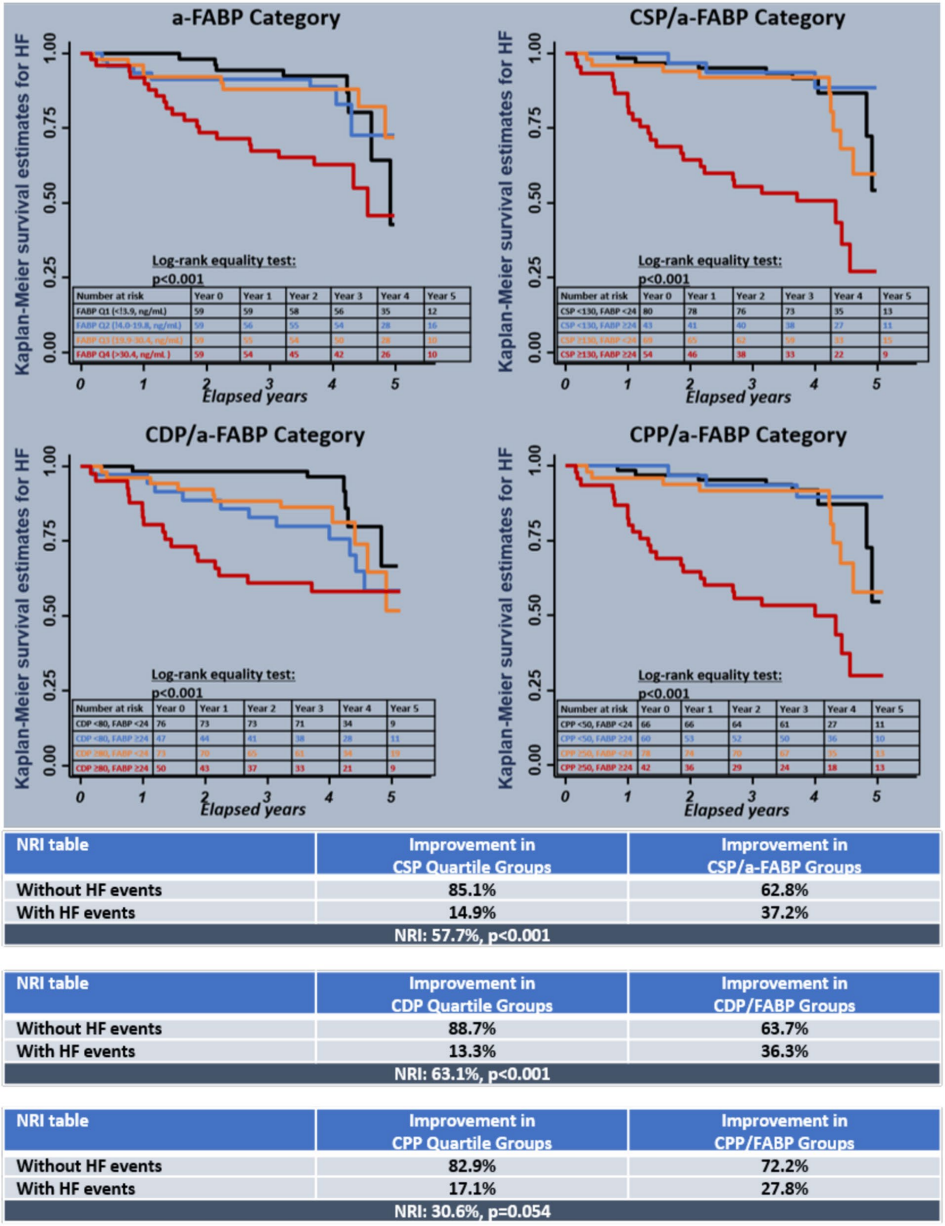
Kaplan–Meier survival analyses exhibited event free survival curves of incident HF when subjects were divided into a-FABP for four quartiles: CSP (130 mm-Hg) vs. a-FABP (24 pg/mL), CDP: (80 mm-Hg) vs. a-FABP (24 ng/mL), and CPP (50 mm-Hg) vs. a-FABP (24 pg/mL).

Kaplan-Meier survival analyses (Fig. 4) showed that incident HF rates were highest in the group of patients with the highest a-FABP levels (HR: 3.08 95% CI 1.38–6.90, p = 0.006, for 4th vs 1st quartile). Figure 4 shows survival curves generated with a-FABP and central hemodynamic indices (CSP/CDP cutoffs 130/80 mmHg, CPP cutoff 50 mmHg, a-FABP cutoff 24 ng/mL). Worsened clinical outcomes were found in patients within both the highest a-FABP and central hemodynamic categories. Combined CSP/a-FABP or CDP/a-FABP data improved HF risk classification versus central hemodynamic categories alone (Fig. 4).

## Discussion

Our current study has several major findings. First, in a study population with known cardiometabolic and HFpEF history, those that experienced future HF events had higher central hemodynamic indices (CSP, CDP, CPP and AIx) and a higher a-FABP level. Second, we observed that a higher a-FABP level was tightly associated with greater central blood pressure and aortic stiffness (i.e., higher CSP, CPP, and AIx), adverse cardiac remodeling, and more impaired cardiac function (including lower TDI-e′, higher E/TDI-e′, and worse GLS). Third, higher baseline a-FABP and greater central aortic stiffness are independent predictors of clinical HF events in models including known prevalent HF history. Incorporation of a-FABP levels into the HF risk classification models based on central hemodynamics improved the accuracy of the models.

### Central hemodynamics and HF risks

Prior reports have shown that resting diastolic functional indices were neither sensitive nor specific enough to identify exertional dyspnea from cardiac causes in patients with HFpEF^27^. As patients with HFpEF demonstrated certain featured hemodynamic and pulsatile abnormalities^28^, unfavorable central hemodynamics reflecting impaired vasodilatory reserve in response to exercise may lead to more impaired LV diastolic filling and decreased myocardial compliance, which likely indicates a more specific pathophysiological role in the exercise intolerance of HFpEF^21,28–30^. A cut-off value of ≥130 mmHg for the central aortic pressure has been recommended for the diagnosis of HTN and is thought to be more cost-effective than diagnoses made with conventional cuff brachial blood pressures^31^. We found that CSP and CPP may serve as predictors of HF events in our high clinical risk cohort, with a clinical cut-off of 130 and 50 mmHg, respectively. Our findings are consistent with those of prior reports regarding the predictive values of central blood pressures for major CV events and mortality in various cohorts^32–35^.

### Associations of a-FABP with central hemodynamics

As mentioned above, metabolic syndrome is closely associated with the development of arterial stiffness, HF, and cardiovascular deaths^2,3^. Interestingly, higher circulating levels of members of the fatty acid-binding protein (FABP) family have been shown to be tightly associated with aging and several metabolic phenotypes in the general population^18,19^. Higher cardiometabolic risks (i.e., obesity, arterial HTN, dyslipidemias, and DM) may all contribute to microvascular pathology or endothelial dysfunction, which is the central pathogenesis of HFpEF, through pro-inflammatory signaling^36–39^. In addition to its role in the pathogenesis of atherosclerosis and CAD^40,41^, a-FABP has also been shown to contribute to microvascular or endothelial dysfunction by stimulating fatty acid-mediated endothelial toxicity through multifaceted mechanisms related to obesity and metabolic disorders; these may include diminished endothelial nitric oxide synthase (eNOs) production, oxidative stress, pro-inflammatory cytokine secretion, and the activation of the renin-angiotensin system and apoptosis^42,43^. Furthermore, Tseng, et al. showed that serum A-FABP positively correlated with aortic arterial stiffness in diabetic patients^13^. Taken collectively, our findings suggested that higher a-FABP levels likely plays a role in modulating altered vascular arterial properties and thereby complicates increased aortic stiffness, which could be detrimental to the heart^13,44^. The hypothetical free fatty-acid mediated vascular toxicity of a-FABP was further supported by the independent relationships between a-FABP and CSP/CPP in this work.

### Associations of a-FABP with cardiac structure and function

**Figure 5:**
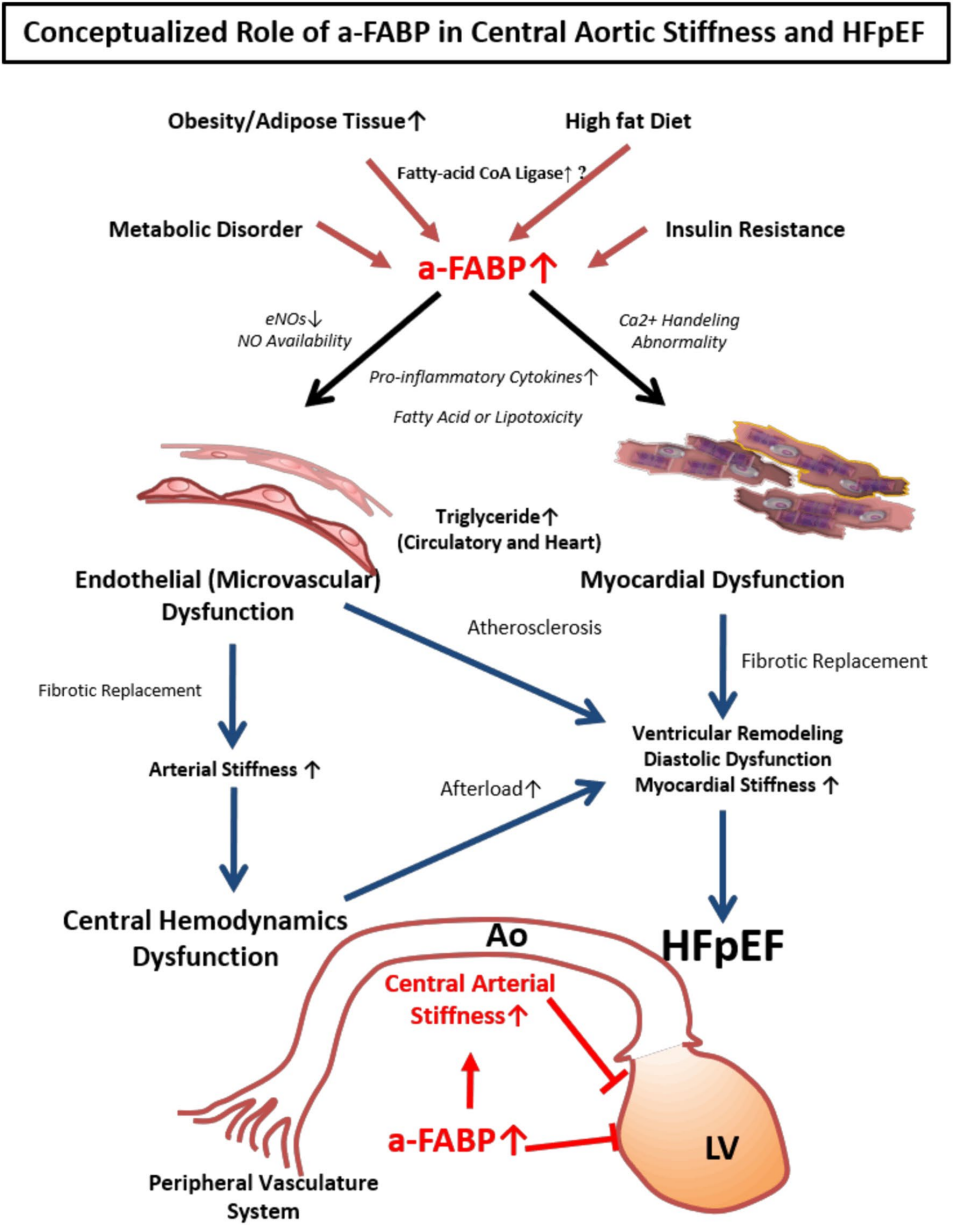
Role of a-FABP in the pathophysiology of heart failure. The hypothetical mechanistic links underlying higher a-FABP, micro-vascular dysfunction, and HFpEF are shown. Heightened central aortic stiffness and impaired myocardial function are key mediators in this pathological process. Ao, aorta, LV, left ventricle. Other abbreviations as Table 1.

In addition to its possible influences on central hemodynamics, higher a-FABP levels may also contribute to the development of HFpEF via distinct pathophysiology. For example, higher circulating a-FABP has been shown to correlate with adverse cardiac remodeling^45^. Liu et al. reported that a-FABP-related cardiac remodeling and cardiac dysfunction may contribute to the development of HF^14^. The influence of a-FABP on HFpEF pathophysiology may likely occur through an alternative yet important pathway. For example, a-FABP has been shown to suppress cardiomyocyte contraction through altered L-type intracellular Ca^2+^ handling and fatty acid toxicity^46^. Though cardiometabolic risk factors, such as a high fat diet, obesity/adipose tissue, metabolic disorder, and insulin resistance may result in increased a-FABP levels, interestingly, we observed that a-FABP levels in our HFpEF population was considerably higher than in those manifesting higher metabolic risks. Indeed, higher a-FABP in our current study was tightly correlated with more impaired myocardial diastolic function and longitudinal myocardial strain, a novel index for subclinical systolic dysfunction and HF beyond chamber-level function (i.e., LVEF)^47^. Given these associations, we speculated that a-FABP may therefore serve as an alternative predictor of HFpEF though mechanisms beyond the effects of arterial function. Abnormal a-FABP displayed inflammation or lipotoxicity-associated endothelial dysfunction and atherosclerosis. Due to the mechanism of advanced myocardial dysfunction in the heart, myocardial stiffness, diastolic dysfunction, and abnormal central hemodynamic parameters were significantly reflected in these HFpEF patients (see the summary in Fig. 5).

### Strengths and limitations

Due to the phenotypic heterogeneity of HFpEF, target-identification and mechanism-of-action studies may have important roles in the diagnosis and therapeutic intervention of HFpEF. Therefore, deep phenotyping is required for the etiologic evaluation and outcome stratification in managing HFpEF. To our best knowledge, our study is the first to explore insights into the pathogenesis of HFpEF from increased central arterial stiffness related to higher adipocyte free fatty acid-binding protein level in a population manifesting cardiometabolic abnormality. Our present work further extended the findings and can be supplemental to prior study in that elevated a-FABP can play an independent role in central arterial stiffness in HFpEF development of ethnic Asians beyond the effect of myocardial suppression^19^. We further explored the prognostic utilization of HF using a-FABP when incorporated in a risk prediction model with central aortic indices measures. However, there are several limitations. First, the relatively small sample size from single center data may lead to selection bias with potential confounders not taken into consideration from our current findings. However, we evaluated the study outcomes by adjusting multiple important confounders to compensate for the inconsistent demographics. Second, exercise physiology measures, for example, the elicited central vascular stiffness during exercise or postural change as important pathophysiology of HFpEF relating to baseline a-FABP level compared to adequate controls was not assessed^6,7,10^. Furthermore, our data interpretation may be biased and confined to ethnic Asian population. Finally, our findings may not be applicable to patients with HFrEF, and further studies are needed to explore the differential pathological mechanisms among a-FABP and central aortic stiffness in both HFpEF and HFrEF.

## Supplementary information

Supplementary Information.

## Abbreviations

HF: Heart failure
HFpEF: Heart failure with preserved ejection fraction
a-FABP: Adipocyte free fatty acid binding protein
CSP: Central systolic pressure
CDP: Central diastolic pressure
CPP: Central pulse pressure
C_AP: Central augmented pressure
C_Aix: Central aortic augmentation index
BNP: Brain natriuretic peptide
PWV: Pulse wave velocity
ECG: Electrocardiogram
E: E wave
A: Late A wave
TDI: Tissue doppler imaging
GLS: Global longitudinal myocardial strain
CM: Cardiometabolic
TDI-e′: Early myocardial relaxation velocity
LV: Left ventricle
E/TDI-e′: LV filling
HTN: Hypertension
LVEF: Left ventricle ejection fraction
DM: Diabetes
BMI: Body mass index
PPP: Peripheral pulse pressure
SBP: Systolic blood pressure
DBP: Diastolic blood pressure
PPA: Pulse pressure amplification
AP: Augmented pressure
PWA: Pulse wave amplification
LS: Longitudinal systolic myocardial strain
eGFR: Estimated glomerular filtration rate
PIIINP: N-terminal pro-peptide of type III procollagen
ROC: Receiver operating characteristic
AUC: Area under the curve
CI: Confidence interval
IQR: Interquartile range

## References

[1] Sharma K, Kass DA (2014). Heart failure with preserved ejection fraction: mechanisms, clinical features, and therapies. Circ Res..

[2] Savji N, Meijers WC, Bartz TM, Bhambhani V, Cushman M, Nayor M (2018). The association of obesity and cardiometabolic traits with incident HFpEF and HFrEF. JACC Heart Fail..

[3] von Bibra H, Paulus W, St John Sutton M (2016). Cardiometabolic syndrome and increased risk of heart failure. Curr Heart Fail Rep..

[4] Suzuki S, Yoshihisa A, Sato Y, Watanabe S, Yokokawa T, Sato T, Oikawa M, Kobayashi A, Yamaki T, Kunii H, Nakazato K, Suzuki H, Saitoh SI, Ishida T, Takeishi Y (2018). Association between sleep-disordered breathing and arterial stiffness in heart failure patients with reduced or preserved ejection fraction. ESC Heart Fail..

[5] Paniczko M, Chlabicz M, Jamiołkowski J, Sowa P, Szpakowicz M, Łapińska M, Kondraciuk M, Ptaszyńska-Kopczyńska K, Raczkowski A, Szpakowicz A, Kamiński KA (2020). Impact of pulse wave velocity and parameters reflecting android type fat distribution on left ventricular diastolic dysfunction in patients with chronic coronary syndromes. J Clin Med..

[6] Saz-Lara A, Cavero-Redondo I, Álvarez-Bueno C, Notario-Pacheco B, Ruiz-Grao MC, Martínez-Vizcaíno V (2021). The acute effect of exercise on arterial stiffness in healthy subjects: a meta-analysis. J Clin Med..

[7] Roman MJ, Devereux RB, Kizer JR, Lee ET, Galloway JM, Ali T (2007). Central pressure more strongly relates to vascular disease and outcome than does brachial pressure: the Strong Heart Study. Hypertension..

[8] McEniery CM, Cockcroft JR, Roman MJ, Franklin SS, Wilkinson IB (2014). Central blood pressure: current evidence and clinical importance. Eur Heart J..

[9] Reddy YNV, Andersen MJ, Obokata M, Koepp KE, Kane GC, Melenovsky V, Olson TP, Borlaug BA (2017). Arterial stiffening with exercise in patients with heart failure and preserved ejection fraction. J Am Coll Cardiol..

[10] Dorogovtsev VN, Yankevich DS, Goswami N (2021). Effects of an innovative head-up tilt protocol on blood pressure and arterial stiffness changes. J Clin Med..

[11] Song F, Zou J, Song Z, Xu H, Qian Y, Zhu H, Liu S, Guan J, Chen J, Yi H (2020). Association of adipocytokines with carotid intima media thickness and arterial stiffness in obstructive sleep apnea patients. Front Endocrinol (Lausanne)..

[12] Aroor AR, Demarco VG, Jia G, Sun Z, Nistala R, Meininger GA, Sowers JR (2013). The role of tissue Renin-Angiotensin-aldosterone system in the development of endothelial dysfunction and arterial stiffness. Front Endocrinol (Lausanne)..

[13] Tseng PW, Hou JS, Wu DA, Hsu BG (2019). High serum adipocyte fatty acid binding protein concentration linked with increased aortic arterial stiffness in patients with type 2 diabetes. Clin Chim Acta..

[14] Hotamisligil GS, Bernlohr DA (2015). Metabolic functions of FABPs—mechanisms and therapeutic implications. Nat Rev Endocrinol..

[15] Ishimura S, Furuhashi M, Watanabe Y, Hoshina K, Fuseya T, Mita T (2013). Circulating levels of fatty acid-binding protein family and metabolic phenotype in the general population. PLoS One..

[16] Rodríguez-Calvo R, Girona J, Alegret JM, Bosquet A, Ibarretxe D, Masana L (2017). Role of the fatty acid-binding protein 4 in heart failure and cardiovascular disease. J Endocrinol..

[17] Egbuche O, Biggs ML, Ix JH, Kizer JR, Lyles MF, Siscovick DS, Djoussé L, Mukamal KJ (2020). Fatty acid binding protein-4 and risk of cardiovascular disease: the cardiovascular health study. J Am Heart Assoc..

[18] Liu M, Zhou M, Bao Y, Xu Z, Li H, Zhang H (2013). Circulating adipocyte fatty acid-binding protein levels are independently associated with heart failure. Clin Sci (Lond)..

[19] Harada T, Sunaga H, Sorimachi H, Yoshida K, Kato T, Kurosawa K, Nagasaka T, Koitabashi N, Iso T, Kurabayashi M, Obokata M (2020). Pathophysiological role of fatty acid-binding protein 4 in Asian patients with heart failure and preserved ejection fraction. ESC Heart Fail..

[20] Lin J-L, Sung K-T, Lai Y-H, Yen C-H, Yun C-H, Su C-H, Kuo J-Y, Liu C-Y, Chien C-Y, Cury RC, Bezerra HG, Hung C-L (2021). Epicardial adiposity in relation to metabolic abnormality, circulating adipocyte FABP, and preserved ejection fraction heart failure. Diagnostics (Basel)..

[21] Holland DJ, Sacre JW, Leano RL, Marwick TH, Sharman JE (2011). Contribution of abnormal central blood pressure to left ventricular filling pressure during exercise in patients with heart failure and preserved ejection fraction. J Hypertens..

[22] Tsao CW, Lyass A, Larson MG, Levy D, Hamburg NM, Vita JA (2015). Relation of central arterial stiffness to incident heart failure in the community. J Am Heart Assoc..

[23] Vallée A, Yannoutsos A, Zhang Y, Henry-Bonniot G, Protogerou A, Topouchian J (2019). Determinants of pulse pressure amplification in hypertensive and diabetic patients. Hypertens Res..

[24] Janner JH, Godtfredsen NS, Ladelund S, Vestbo J, Prescott E (2010). Aortic augmentation index: reference values in a large unselected population by means of the SphygmoCor device. Am J Hypertens..

[25] Lang RM, Badano LP, Mor-Avi V, Afilalo J, Armstrong A, Ernande L (2015). Recommendations for cardiac chamber quantification by echocardiography in adults: an update from the American Society of Echocardiography and the European Association of Cardiovascular Imaging. J Am Soc Echocardiogr..

[26] Hung CL, Gonçalves A, Lai YJ, Lai YH, Sung KT, Lo CI (2016). Light to moderate habitual alcohol consumption is associated with subclinical ventricular and left atrial mechanical dysfunction in an asymptomatic population: dose-response and propensity analysis. J Am Soc Echocardiogr..

[27] Holland DJ, Prasad SB, Marwick TH (2010). Contribution of exercise echocardiography to the diagnosis of heart failure with preserved ejection fraction (HFpEF). Heart..

[28] Chirinos JA (2017). Deep phenotyping of systemic arterial hemodynamics in HFpEF (part 2): clinical and therapeutic considerations. J Cardiovasc Transl Res..

[29] Weber T, O'Rourke MF, Ammer M, Kvas E, Punzengruber C, Eber B (2008). Arterial stiffness and arterial wave reflections are associated with systolic and diastolic function in patients with normal ejection fraction. Am J Hypertens..

[30] Borlaug BA, Lam CS, Roger VL, Rodeheffer RJ, Redfield MM (2009). Contractility and ventricular systolic stiffening in hypertensive heart disease insights into the pathogenesis of heart failure with preserved ejection fraction. J Am Coll Cardiol..

[31] Cheng HM, Chuang SY, Sung SH, Wu CC, Wang JJ, Hsu PF (2019). 2019 Consensus of the Taiwan Hypertension Society and Taiwan Society of Cardiology on the clinical application of central blood pressure in the management of hypertension. Acta Cardiol Sin..

[32] Jankowski P, Kawecka-Jaszcz K, Czarnecka D, Brzozowska-Kiszka M, Styczkiewicz K, Loster M (2008). Pulsatile but not steady component of blood pressure predicts cardiovascular events in coronary patients. Hypertension..

[33] Pini R, Cavallini MC, Palmieri V, Marchionni N, Di Bari M, Devereux RB (2008). Central but not brachial blood pressure predicts cardiovascular events in an unselected geriatric population: the ICARe Dicomano Study. J Am Coll Cardiol..

[34] Savarese G, Lund LH (2017). Global public health burden of heart failure. Card Fail Rev..

[35] Niiranen TJ, Kalesan B, Mitchell GF, Vasan RS (2019). Relative contributions of pulse pressure and arterial stiffness to cardiovascular disease. Hypertension..

[36] Hajouli S, Ludhwani D. Heart failure and ejection fraction *StatPearls* Treasure Island (FL): StatPearls Publishing Copyright © 2020, StatPearls Publishing LLC.; 2020. 31971755

[37] Arcopinto M, Schiavo A, Salzano A, Bossone E, D'Assante R, Marsico F (2019). Metabolic syndrome in heart failure: friend or foe?. Heart Fail Clin..

[38] Gao WD, Murray CI, Tian Y, Zhong X, DuMond JF, Shen X (2012). Nitroxyl-mediated disulfide bond formation between cardiac myofilament cysteines enhances contractile function. Circ Res..

[39] Altara R, Giordano M, Nordén ES, Cataliotti A, Kurdi M, Bajestani SN (2017). Targeting obesity and diabetes to treat heart failure with preserved ejection fraction. Front Endocrinol (Lausanne)..

[40] von Eynatten M, Breitling LP, Roos M, Baumann M, Rothenbacher D, Brenner H (2012). Circulating adipocyte fatty acid-binding protein levels and cardiovascular morbidity and mortality in patients with coronary heart disease: a 10-year prospective study. Arterioscler Thromb Vasc Biol..

[41] Chow WS, Tso AW, Xu A, Yuen MM, Fong CH, Lam TH (2013). Elevated circulating adipocyte-fatty acid binding protein levels predict incident cardiovascular events in a community-based cohort: a 12-year prospective study. J Am Heart Assoc..

[42] Ghosh A, Gao L, Thakur A, Siu PM, Lai CWK (2017). Role of free fatty acids in endothelial dysfunction. J Biomed Sci..

[43] Aragonès G, Ferré R, Lázaro I, Cabré A, Plana N, Merino J (2010). Fatty acid-binding protein 4 is associated with endothelial dysfunction in patients with type 2 diabetes. Atherosclerosis..

[44] Chen MC, Hsu BG, Lee CJ, Yang CF, Wang JH (2017). High serum adipocyte fatty acid binding protein level as a potential biomarker of aortic arterial stiffness in hypertensive patients with metabolic syndrome. Clin Chim Acta..

[45] von Jeinsen B, Ritzen L, Vietheer J, Unbehaun C, Weferling M, Liebetrau C (2020). The adipokine fatty-acid binding protein 4 and cardiac remodeling. Cardiovasc Diabetol..

[46] Lamounier-Zepter V, Look C, Alvarez J, Christ T, Ravens U, Schunck WH (2009). Adipocyte fatty acid-binding protein suppresses cardiomyocyte contraction: a new link between obesity and heart disease. Circ Res..

[47] Potter E, Marwick TH (2018). Assessment of left ventricular function by echocardiography: the case for routinely adding global longitudinal strain to ejection fraction. JACC Cardiovasc Imaging..

