# Supplementary material for: Association of free fatty acid binding protein with central aortic stiffness, myocardial dysfunction and preserved ejection fraction heart failure
Source: Sci Rep. 2021 Aug 13;11:16501. doi: 10.1038/s41598-021-95534-1 (PMC8363603; doi:10.1038/s41598-021-95534-1)
Supplement: Supplementary file 1 — Supplementary Information. [file 41598_2021_95534_MOESM1_ESM.docx]

**(Supplementary Material)**

***Associations of Central Hemodynamics with Cardiac Structure and Function***

Among 254 study participants, 249 (98.0%) had a conventional echocardiography assessment for LV geometry and LV mass and 246 (96.8%) had TDI-based measurements. Higher CSP was associated with greater LVM index, lower TDI-s’ and TDI-e’, higher E/TDI-e’, and worse GLS (r=0.19, -0.28, -0.36, 0.44, and -0.23, respectively, all p<0.001). The associations between higher CDP and greater LVMi, lower TDI-e’, and higher E/TDI-e’ were attenuated, though still significant (r=0.18, -0.20, 0.17, and -0.17, respectively, all p<0.05).Higher CPP was associated with lower TDI-s’ and TDI-e’, higher E/TDI-e’, and worse GLS (r=-0.32, -0.30, -0.43, and -0.16, respectively, all p<0.05). After controlling for baseline co-variates and traditional blood pressure components (SBP, DBP, and PP in CSP, CDP, and CPP models, respectively), higher central hemodynamic indices were significantly associated with lower TDI-e’ and higher E/TDI-e’ (Table 2). Higher CSP, was marginally associated with higher BNP levels in a multivariate model (adjusted coefficient: 6.73, 95% CI: -1.1-14.5, p=0.09), but CDP and CPP were not. Higher SBP and PP were associated with greater LVMi and BNP in the fully adjusted models, though not with other cardiac functional indices (all non-significant, data not shown).

**Supplemental Figure 1:**

**
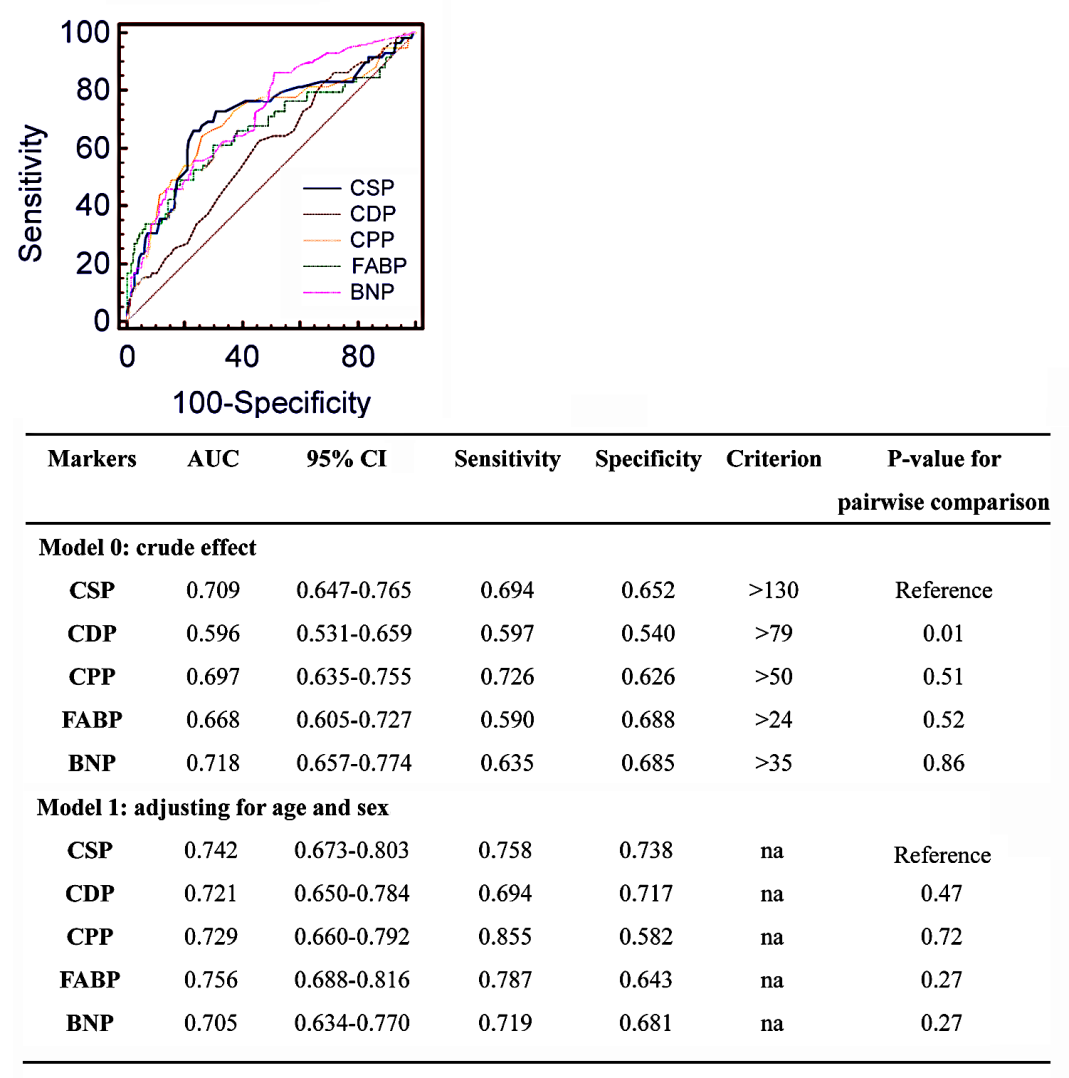
**

FABP indicates a-FABP.

Both CSP (C-statistics: 0.71, 95% CI: 0.65-0.77) and CPP (C-statistics: 0.70, 95% CI: 0.64-0.76) had higher prognostic power than CDP (AUC: 0.60, 95% CI: 0.53-0.66) for HF events (Supplement Figure 1). Higher C_AP (C-statistics: 0.58, 95% CI: 0.50-0.67, OR: 1.02, p=0.13) and higher C_Aix (C-statistics: 0.61, 95% CI: 0.52-0.67, OR: 1.04, p=0.028) both exhibited modest prognostic values for HF incidence. Criterion values presented indicated the optimal threshold value (cutoff point) of various parameters in predicting HF events calculated using the Youden index. The individual Youden index of CSP, CDP, CPP a-FABP and BNP was 0.35, 0.14, 0.35, 0.35 and 0.32, respectively.

**Supplemental Table 1: Associations of central hemodynamic indices with key cardiac structural and functional parameters**

| **Model 1 (+ clinical co-variates)** | **CSP (+10mmHg)** | | **CDP (+10mmHg)** | | **CPP (+10mmHg)** | |
| --- | --- | --- | --- | --- | --- | --- |
|  | Coef. (95% CI) | p value | Coef. (95% CI) | p value | Coef. (95% CI) | p value |
| Left ventricular mass index, gm/m^2^* | 1.08 (-0.01, 2.17) | 0.051 | 0.74 (-1.23, 2.70) | 0.46 | 1.4 (0.03, 2.85) | 0.045 |
| TDI-e’ (average), cm/sec | -0.21 (-0.32, -0.11) | <0.001 | -0.33 (-0.53, -0.14) | 0.001 | -0.19 (-0.33, -0.04) | 0.01 |
| E/TDI-e’ (average) | 0.55 (0.36, 0.75) | <0.001 | 0.60 (0.23, 0.96) | 0.001 | 0.62 (0.37, 0.87) | <0.001 |
| TDI-s’ (average), cm/sec | -0.13 (-0.22, -0.05) | 0.002 | -0.14 (-0.29, 0.02) | 0.088 | -0.16 (-0.26, -0.05) | 0.006 |
| GLS, % | -0.16 (-0.31, -0.02) | 0.028 | -0.26 (-0.52, 0.01) | 0.059 | -0.15 (-0.34, 0.05) | 0.14 |
| **Model 2 (+ traditional BP variables)** | **CSP (+10mmHg) + SBP** | | **CDP (+10mmHg) + DBP** | | **CPP (+10mmHg) + PP** | |
|  | Coef. (95% CI) | p value | Coef. (95% CI) | p value | Coef. (95% CI) | p value |
| Left ventricular mass index, gm/m2* | 0.63 (-0.54, 1.80) | 0.29 | -0.44 (-2.60, 1.72) | 0.69 | 1.13 (-0.30, 2.55) | 0.12 |
| TDI-e’ (average), cm/sec | -0.20 (-0.32, -0.08) | 0.001 | -0.30 (-0.52, -0.08) | 0.007 | -0.16 (-0.31, -0.02) | 0.025 |
| E/TDI-e’ (average) | 0.56 (0.36, 0.77) | <0.001 | 0.55 (0.15, 0.95) | 0.008 | 0.59 (0.34, 0.85) | <0.001 |
| TDI-s’ (average), cm/sec | -0.13 (-0.22, -0.04) | 0.005 | -0.11 (-0.28, 0.06) | 0.2 | -0.15 (-0.26, -0.03) | 0.01 |
| GLS, % | -0.14 (-0.30, 0.02) | 0.08 | -0.21 (-0.50, 0.09) | 0.17 | -0.12 (-0.32, 0.08) | 0.23 |

*****in which model body mass index was not entered. Abbreviations: CSP, central systolic pressure; CDP, central diastolic pressure; CPP, central pulse pressure; TDI-e’, peak myocardial diastolic relaxation velocity on tissue-based Doppler; E/TDI-e’, E wave/TDI-e’; TDI-s’, peak myocardial systolic relaxation velocity of tissue-based Doppler; GLS, global LV longitudinal systolic myocardial strain.
